# Probing the structural, mechanical, phonon, thermal, and transport properties of magnetic halide perovskites XTiBr_3_ (X = Rb, Cs) through ab-initio results

**DOI:** 10.1038/s41598-023-34047-5

**Published:** 2023-06-05

**Authors:** Vishal Shivhare, Saveer Ahmad Khandy, Dinesh C. Gupta

**Affiliations:** 1 Condensed Matter Theory Group, School of Studies in Physics, Jiwaji University, Gwalior, 474011 India; 2 Condensed Matter Theory Group, School of Studies in Physics, Jiwaji University, Gwalior, 474011 India

**Keywords:** Materials science, Physics

## Abstract

Herein, we have first reported the intrinsic properties, including structural, mechanical, electronic, magnetic, thermal, and transport properties of XTiBr_3_ (X = Rb, Cs) halide perovskites within the simulation scheme of density functional theory as integrated into *Wien2k*. First and foremost, the structural stability in terms of their ground state energies has been keenly evaluated from their corresponding structural optimizations, which advocate that XTiBr_3_ (X = Rb, Cs) has a stable ferromagnetic rather than the competing non-magnetic phase. Later on, the electronic properties have been computed within the mix of two applied potential schemes like Generalized Gradient Approximation (GGA) along with Trans-Bhala modified Becke Johnson (TB-mBJ), which thoroughly addresses the half-metallic behaviour with spin-up as metallic and in contrast to opposite spin-down channel signatures the semiconducting behaviour. Furthermore, the spin-splitting seen from their corresponding spin-polarised band structures offers a net magnetism of 2 µB which lends their opportunities to unlock the application branch of spintronics. In addition, these alloys have been characterised to show their mechanical stability describing the ductile feature. Moreover, phonon dispersions decisively certify the dynamical stability within the density functional perturbation theory (DFPT) context. Finally, the transport and thermal properties predicted within their specified packages have also been forwarded in this report.

## Introduction

In the present era, solid-state materials, with their better and excellent properties, are recognized to be essential candidates for the advancement of new technological applications. Similarly, halide perovskites (HPs) have been extensively studied recently. Till now, considerable interest has been paid due to their broad significance in optoelectronics, sensors, high-capacity memory cells, substrates, electrodes in fuel cells, and prominent sources for spintronic applications^1^. The reason for having this can be attributed due to their chemical flexibility and tunability, which involves every element within their lattice structures. Keeping in view, magnetism, as one of the prominent properties related to these perovskites, has transformed a complete spectrum of technologies, such as biomedical imaging, and continues to bring forth to find suitable applications in compatible spintronics. A bit of overview on spintronics is a relatively new research area that exploits the spin-degree of freedom and its fundamental charge for transporting data for longer distances and has influenced various technological fields^2^. It also aims to modify the spin degree to achieve faster transport and dissipation-less properties^3–5^. However, some long-term challenges must be addressed, including spin-polarized carrier synthesis, spin injection, spin manipulation/detection, and long-distance spin-polarized transport. In this regard, half-metals^6,7^, magnetic semiconductors^8,9^, topological insulators^10^, and spin-gapless semiconductors^11^ are the materials that have been keenly analysed to resolve the mentioned issue. But on behalf of these materials, their small quantity of spin-polarized carriers and low Curie temperature are two critical hindrances before realizing them for such issues.

However, halide perovskite alloys are the spotlight for spin transportation due to compositional adaptability and are in high demand for such features. In addition, the research has been further extended to see the multidimensional functionalities of these alloys and to understand the various phenomena exhibited within their lattice structures, such as magnetoresistance^12^, ferromagnetism^13,14^, magneto-optics^15^, and metallicity^16^. However, at the Fermi-Level (E_F_), they display a prominent spin-magnetic behaviour thus, promote 100% spin-polarisation^17–19^. More likely, these materials can transform the squandered (waste) heat into a valuable form of electrical energy without leaving any harmful by-products^20,21^. Therefore, paving their route for thermoelectric applications^22–24^. The present study investigates the structural, mechanical, and thermal properties of newly magnetic halide perovskites XTiBr_3_ (X = Rb, Cs). The ground-state features and possible application of these halides have not yet been examined in the scientific literature. Hence, we performed DFT-based simulations thoroughly to examine the structural stability, mechanical stability, electronic profile, phonon dispersions, and transport coefficients of these perovskites.

## Computational details

The structural, electronic, and magnetic properties of the cubic perovskites RbTiBr_3_ and CsTiBr_3_ have been significantly carried out by solving the Kohn Sham equations within the framework of density functional theory as integrated into *Wien2k*^25^. The calculations of these materials primarily acted upon the inclusion of GGA^26^. In GGA formulism, the exchange–correlation (*E*_*xc*_) is viewed as a derivative of the local charge density and the associated gradient. However, GGA often fails to give exact band profiles for the systems containing *d/f* electrons because of the self-interaction effect and insufficient potential for highly localized states. Accordingly, the GGA method needs to be complemented with other modified approximations to express the properties of such systems more precisely. Thus, the mBJ^27^ has been adopted to envisage the precise treatment of highly correlated *d*/*f *electron systems as it is reasonably effortless and ab-initio. Because of this, the FP-LAPW approach splits the crystal space into non-overlapping muffin tin spheres with R_MT_ as the radius of muffin tin (MT) sphere and regions beyond the muffin tin spheres known as interstitial regions that are subjected to a plane-wave basis set for eigenvalue convergence. For non-overlapping atomic spheres, the linearized augmented plane-wave basis set with *l*_max_ = 10 and R_MT_K_max_ = 7 (K_max_ denotes the highest possible *k*-value, and R_MT_ is the radius of the smallest sphere) has been adopted to ensure charge and energy convergence. Fourier series expansion of charge density and potential was made within the interstitial region with the wave vector up to G_max_ = 12 a.u^−1^. The tetrahedral method^28^ and a dense *k*-mesh of 4000 k-points within the Monkhorst and Pack convention scheme were used. The threshold energy of -6.0 Ry is selected as the cut-off for separating the core and valence states. To ensure the accuracy of self-consistent calculations, the convergence tolerance for charge and energy is selected to be 10^−4^ e and 10^−4^ Ry, respectively. The thermoelectric response has been explored with the help of semi-classical Boltzmann theory as embedded in the BoltzTraP code^29^. The mechanical behaviour of these alloys has been probed using the elastic constant calculations by the cubic-elastic package^30^. The thermal properties have been assessed using the Gibbs2 package in aggregation with the *Wien2k* code^31^.

## Results and discussions

The spin-polarized density functional theory (DFT) has been systematically utilized to figure out the various physical properties of XTiBr_3_ (X = Rb, Cs). Their detailed summary has been keenly addressed under the following sub-headings.

### Structural properties

**Figure 1:**
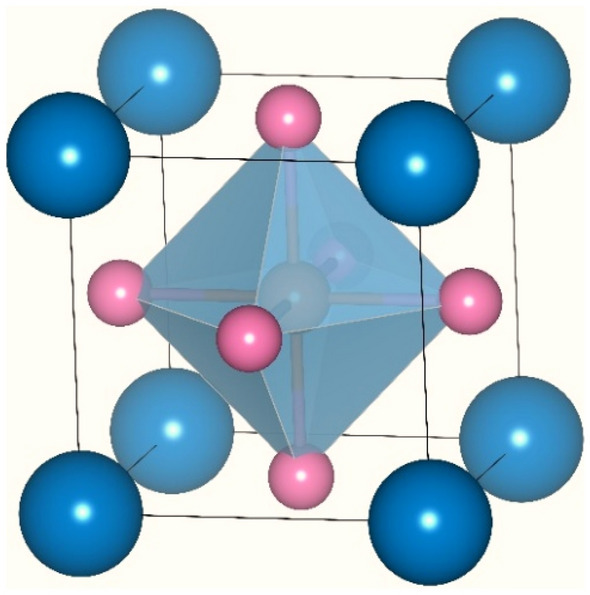
Molecular crystal structure of XTiBr_3_ (X = Rb, Cs); here, the dark blue sphere () represents Rb and Cs, the pink sphere () Br, and the red sphere () Ti.

The crystal structure of XTiBr_3_ (X = Rb, Cs) halide perovskites resemble the ordered cubic lattice structure with space symmetry Pm-3m. The lattice parameters (a = b = c) are equal in these crystal structures, and the crystallographic angles are 90°. Interestingly, the corresponding atoms of these molecular crystal structures XTiBr_3_ (X = Rb, Cs) where the monovalent Rb/Cs cations having 12-fold coordinated with Bromine anions are allocated at the origin with coordinates (0, 0, 0), The pentavalent Ti cations lie within oxygen octahedral occupying the body-center place (0.5, 0.5, 0.5). The oxygen atoms hold the face center positions (0.5, 0.5, 0) of the cubic unit cell as visualized in Fig. 1. So, the arrangement of atoms within their own specified positions defines the complete geometry, allowing us to see the next steps in structural stability in terms of their ground state energies.

To obtain the total ground state energy of these two perovskite systems, we have significantly carried the structural optimization by performing a least-squares fit of the crystal energy against the unit cell volume using the Birch-Murnaghan equation of state^32^ in distinct ferromagnetic (FM) and non-magnetic (NM) phases expressed as:

1 $$E(V) = E_{0} + \frac{{9B_{0} V_{0} }}{16}\left\{ {\left[ {\left( {\frac{{V_{0} }}{V}} \right)^{2/3} - 1} \right]} \right.B^{\prime}_{0} + \left[ {\left( {\frac{{V_{0} }}{V}} \right)^{2/3} - 1} \right]^{2} \left. {\left[ {6 - 4\left( {\frac{{V_{0} }}{V}} \right)^{2/3} } \right]} \right\}.$$

**Figure 2:**
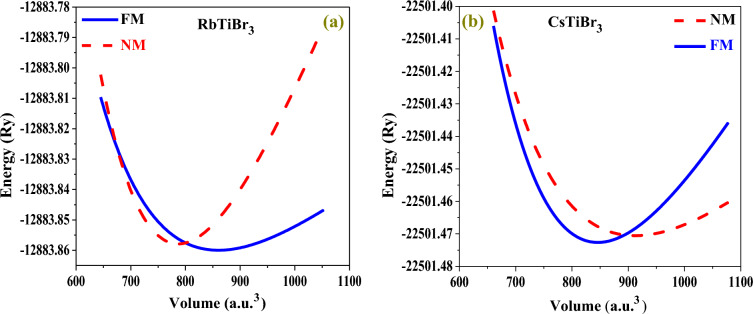
(**a**,**b**) Energy (*Ry*) vs. volume (*a.u*.^3^) structural relaxation of halide perovskites.

The terms *E*(*V*), *V*, and *B*_*0*_ (*B'*_*0*_) in this equation stand the ground state energy, unit cell volume, and the bulk modulus (derivate of the bulk modulus), respectively. However, from the resulting structural relaxation, it is seen that the two crystal structures favour the release of the least amount of energy in the FM phase rather than the competing non-magnetic phase, as displayed in Fig. 2a,b.

Therefore, the amount of energy released from their corresponding lattice structures hints at the perfect occurrence of these materials if synthesised experimentally. It also indicates that these alloys are structurally stable regarding their ground state energies. Therefore, on behalf of this, specific relaxed parameters inclusive of lattice constant (***a***_***0***_), volume (**V**_**0**_), bulk modulus (**B**_**0**_), derivative of bulk modulus (***B***'_**0**_), and energy (***E***_***0***_) have been fetched. These parameters are in satisfactory agreement with previously published computational and experimental findings, as shown in Table 1, thus validating our results.

**Table 1:** The physical ground state properties of XTiBr3 (X = Rb, Cs) estimated in stable FM configurations.

Crystals	*a*_*0*_ (Å)	*V*_*o*_ (a.u.^3^)	B_*0*_ (GPa)	*B'*_*0*_	*E*_*o*_ (Ry)	*t*	E_coh_
Rb^+1^Ti^+2^Br^−1^_3_	5.03	860.15	14.38	5.88	− 12,883.85	0.95	2.58
Cs^+1^Ti^+2^Br^−1^_3_	5.13	912.04	14.53	5.69	− 22,501.47	0.96	2.51

Furthermore, the structural stability of XTiBr_3_ (X = Rb, Cs) has been explored in terms of the well-known Goldschmidt's tolerance factor (*t*)^33^. The tolerance factor of these alloys has been obtained by the ionic radii method and can be written as;

2 $$t = 0.707\frac{ < A - O > }{{ < B - O > }} = 0.707\frac{{(r_{A} + r_{O} )}}{{(r_{B} + r_{O} )}}.$$

 < Rb/Cs − Br > and < Ti − Br > are the average bond lengths between Rb/Cs-Br and Ti-Br, respectively. Therefore, adopting the values of each atom in the above-mentioned equation results in the value of* t,* which perfectly lies in between the range of 0.93–1.04 as displayed in Table 1 hence, supports the cubic structural stability of these two halide systems. Concerning this, the structural stability of these two perovskites has been extended by illustrating their cohesive energies, which infers that a large amount of energy is supplied to knock an atom from its attractive surroundings.

### Mechanical properties

The elastic properties provide a better understanding of the material’s mechanical properties and thus signify their route towards industrial and various important technological purposes. However, it also describes the ability of a material to recover its original shape over the elimination of external forces acting on it. Similarly, for the case of RbTiBr_3_ and CsTiBr_3_ perovskite alloys, we have taken advantage of Charpin's Cubic-elastic package^30^ as integrated into the *Wien2k* to calculate the elastic features. More likely, evaluating these constants depends upon the material’s symmetry. Therefore, for the RbTiBr_3_ and CsTiBr_3_ cubic systems, just three stiffness constants, *C*_11_, *C*_12_, and *C*_44_, must define their elastic stability. The elastic constant *C*_11_ reasonably describes the longitudinal compression and is related to the hardness of the material. *C*_12_ refers to the transverse distortion correlated with the Poisson’s ratio and is dependent upon B-site cation, whereas *C*_44,_ the elastic parameter, is linked with the Shear modulus. Since all the values calculated for these alloys are positive, as mentioned in Table 2**,** satisfies the generalized Born-Huang stability conditions also^34,35^, i.e.,

3 $$\left( {{\text{C}}_{{{11}}} > \, 0} \right), \, \left( {{\text{C}}_{{{12}}} > \, 0} \right), \, \left( {{\text{C}}_{{{44} }} > \, 0} \right),{\text{C}}_{{{11}}} + {\text{ 2C}}_{{{12}}} > \, 0,{\text{ C}}_{{{11}}} - {\text{C}}_{{{12}}} > \, 0,{\text{ C}}_{{{44}}} > \, 0$$

describes the elastic stability of these alloys. Further on addressing the mechanical stability, we have supplied all these elastic values in different mathematical expressions to get the value of bulk modulus (B), Young’s modulus (Y), and shear modulus (G). The bulk (B) and shear moduli (G) have been derived by using the Viogt-Reuss-Hill approximations^36–38^. Subsequently, the characteristic feature of defining whether these materials are ductile or brittle can be analyzed from Pugh Ratio (B/G), Poisson’s ratio (ʋ), Cauchy’s pressure (C_P_), and Zener’s Anisotropy (A) as enumerated by the following equations.

4 $$B_{V} = B_{G} = B = \frac{{(C_{11} + 2C_{12} )}}{3\,},$$

5 $$G_{V} = \frac{{(C_{11} - C_{12} + 3C_{44} )}}{5\,};\,\,\,\,G_{R} = \frac{{5(C_{11} - C_{12} )C_{44} }}{{4C_{44} + 3(C_{11} - C_{12} )}};\,\,\,\,\,\,G = \frac{{G_{V} + G_{R} }}{2},$$

6 $${\text{ Y = }}\frac{9BG}{{3B + G}};\,\,\,\,\,\,\upsilon { = }\frac{3B - Y}{{6B}}{; }\,\,\,\,A \, = \, \frac{{2C_{44} }}{{\left( {C_{11} - C_{12} } \right)}}\,\,\,{\text{and}}\,\,\,{\text{C}}_{{\text{P}}} = {\text{ C}}_{{{12}}} - {\text{C}}_{{{44}}} .$$

**Table 2:** Derived mechanical parameters of RbTiBr_3_ and CsTiBr_3_ alloys respectively.

Parameter		RbTiBr_3_	CsTiBr_3_
Elastic constants	*C*_11_ (GPa)	85.08	87.03
	*C*_12_ (GPa)	44.87	36.53
	*C*_44_ (GPa)	26.22	26.16
Bulk modulus (B in GPa)		58.27	53.37
Young’s modulus (Y in GPa)		62.33	66.65
Shear modulus (G in GPa)		23.58	25.79
Pugh ratio (B/G in GPa)		2.47	2.06
Poisson’s ratio (ʋ) (in GPa)		0.32	0.29
Cauchy’s pressure (C_P_ in GPa)		40.21	50.5
Zener’s anisotropy factor (A)		1.30	1.04

The description of the above-specified parameters can be expressed by taking the Bulk modulus (B) first, which explains the volumetric change caused by the external pressure implemented on these materials, thus foretells the compressible or incompressible nature depending upon the volumetric change. If a material experiences less change in its volume, then it possesses incompressible nature and vice versa. Herein, in the case of these two alloys, RbTiBr_3_ and CsTiBr_3_ show incompressible natures. Another noteworthy property related to these compounds is Young’s modulus (Y) which significantly displays their suitable applications in industrial and aerospace engineering technologies. The calculated values of Young’s modulus manifest that the stiffness character is preserved within these materials.

In the same way, the shear modulus (G) illustrates the transverse deformation. However, the characteristics of these materials in terms of ductility or brittleness can be achieved primarily from Pugh’s ratio denoted by (B/G)^39^. The critical value of B/G is 1.75. According to Pugh, if a material has a value greater than the critical value, i.e., 1.75, it can be considered ductile. The value below it indicates the brittle nature. So, for the present alloys, the value of B/G is 2.47 and 2.06, respectively; more significant than 1.75 features a ductile rather than brittle nature. Also, Poison’s ratio (ʋ)^40^ is used in practice to predict the ductile or brittle nature of the materials. The obtained value of Poison’s ratio (ʋ) is 0.32 for RbTiBr_3_ and 0.29 for CsTiBr_3_ examines the ductile nature as both the values possessed by the alloys are more significant than 0.25. Similarly, the calculated value of Cauchy’s pressure (C_P_)^41^ labels the exact ductile nature. In addition, Zener’s anisotropic factor (A) reveals the isotropic and anisotropic nature of the compound. Suppose the value of (A) is equivalent to unity. In that case, the compound is purely isotropic, which means the material’s properties are identical in the exact directions and vice versa. From the estimated data, the value of (A) is greater than 1 signifies the anisotropic nature of these alloys, and all these values have been enlisted in Table 2.

As both alloys are anisotropic in nature, therefore, the sound velocities will be examined in different directions having different velocities. The cubic symmetry of the present alloy suggests that pure modes of elastic waves can only exist in [100], [110], and [111] directions. The calculated value of phase velocity is given in Table 3; with longitudinal and transverse velocity, one can determine the Debye temperature (**θ**_**D**_). The Debye temperature of a material can be estimated by a classical method using mean sound velocity^42^, which expresses the temperature at which the collective behaviour of the atoms usually tends to the independent mode of vibration. In addition, the most critical thermodynamic quantity called melting temperature (T_m_)^43^ of these halide alloys has been evaluated by using the following equation;

7 $${\text{T}}_{{\text{m}}} = \, \left[ {{553}\left( {\text{K}} \right) \, + \, \left( {{5}.{911}} \right){\text{ C}}_{{{11}}} } \right]{\text{ GPa }} \pm { 3}00\,{\text{K}}{.}$$

**Table 3:** Calculated sound (m/s), averaged velocities, Debye temperature (K), and melting temperature (K) for RbTiBr_3_ and CsTiBr_3._

[100]			[110]			[111]			*v*_*l*_	*v*_*t*_	*v*_*m*_	θ_D_	T_m_
*v*_*l*_	*v*_*t1*_	*v*_*t2*_	*v*_*l*_	*v*_*t1*_	*v*_*t2*_	*v*_*l*_	*v*_*t1*_	*v*_*t2*_					
2.41	1.34	1.34	2.31	1.66	1.34	2.53	1.23	1.23	6075	3115	810	300.48	1055.90
2.36	1.30	1.30	2.19	3.25	1.30	2.38	1.28	1.28	2377	1853	456	352.39	1067.46

The melting temperature values are 1055.90 K for RbTiBr_3_ and 1067.46 K for CsTiBr_3_. Here, these values describe the retention of lattice structure and clarify that these alloys do not go into another phase transformation. So, concisely, we believe that the reported values of mechanical properties may provide substantial proof for their use in various engineering and industrial applications and provide verification of fabricating these materials experimentally.

### Phonon stability

**Figure 3:**
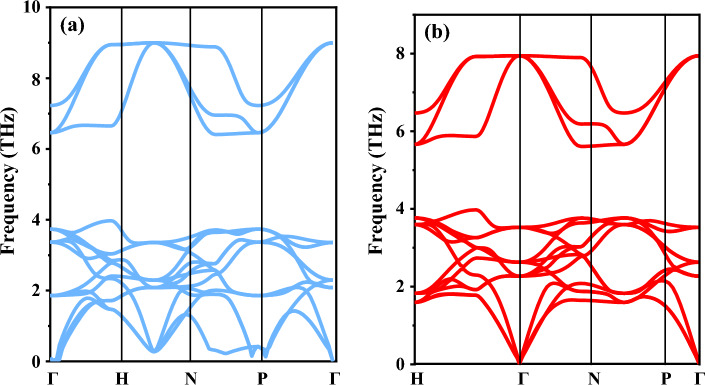
(**a**,**b**) Phonon dispersion spectrum for RbTiBr_3_ and CsTiBr_3_ perovskites alloys.

Phonon dispersions are essential to study the Raman vibrational spectroscopy of materials^44^. Some of the physical characteristics, inclusive of electrical and thermal conductivity, are significantly influenced by it. In this computational aspect, while executing the dynamical context of RbTiBr_3_ and CsTiBr_3_ perovskites within their primitive unit cells, we have rigorously used the pseudopotential computation of Quantum Espresso^45^ in which density functional perturbation theory (DFPT) is embedded within it. The 15 phonon branches resulting from the five constituent atoms, including three acoustic with zero frequency at Γ-point corresponds to *k* = 0 in reciprocal space and rest of the other twelve frequencies are non-zero, called optical phonons as shown in Fig. 3a,b. Moreover, acoustic branch consists of two longitudinal aquatic (LA) and one transverse (TA) mode. Subsequently the acoustic branches arise due the vibration of heavier Cs atom, while optical branches are seen through the vibration of lighter atom. The intermediate branches are displayed by the rigorous vibration of Ti. However, in a number of high-symmetry crystals, and along the high-symmetry directions, the atomic vibrations are either polarized along the propagation wave vector *k*, or perpendicular to *k* according to factor theory; the optical branches can be highly described by the combination of Raman, infrared and silent modes depending upon the frequency range. However, it is analyzed that the absence of negative frequencies legitimates the dynamic context of these simple cubic halide structures.

### Electronic properties

**Figure 4:**
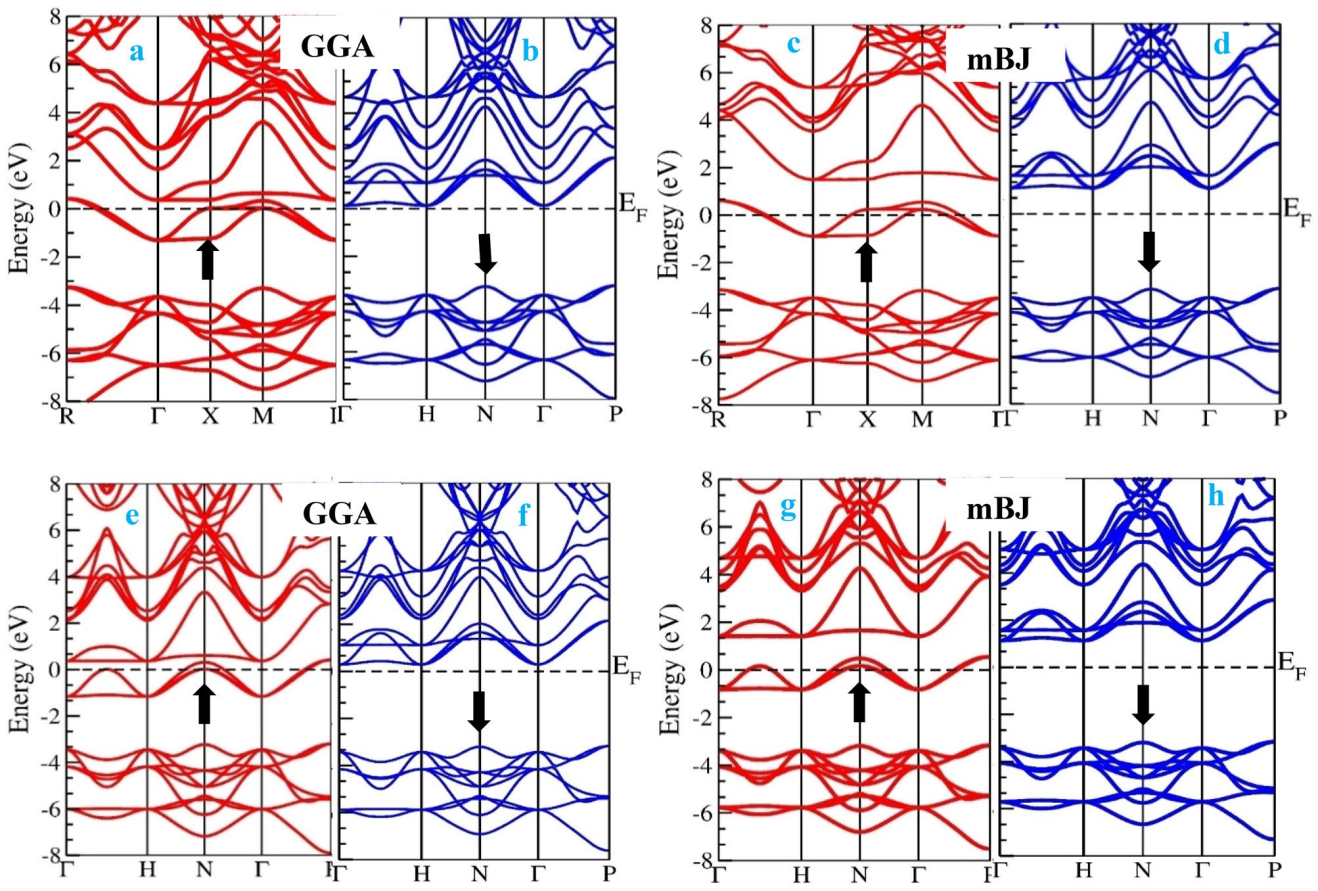
(**a**–**d**) Spin-polarized band structures of RbTiBr_3_ in GGA and mBJ potential scheme. (**e**–**h**) Spin-polarized band structures of CsTiBr_3_ in GGA and mBJ potential scheme.

We performed the electronic structure calculations of RbTiBr3 and CsTiBr3 perovskite systems using the density functional theory. Primarily, the balanced lattice parameter retrieved from the Brich-Murnaghan equation has been utilized to evaluate the electronic structures of these alloys within the GGA approximation scheme. GGA potential provides satisfactory results for binary (s,p) systems but lags to execute the electronic structures of materials containing d/f electrons, which is considered a short-range potential. Because of this lagging performance, we have adopted a sophisticated method known as mBJ with its accuracy of providing results closer to the experimental results. However, the insertion of these two approximation schemes (GGA and mBJ) over these alloys depicts the spin-polarized half-metallic nature, as displayed in Fig. 4a–h. The half-metallic nature can be addressed as these materials exhibit metallic character in one spin channel and contrast to opposite spin divulges semiconducting behavour. Individually for the case of RbTiBr_3_, the band gap within the approximation scheme of GGA results and equals 3.3 eV in spin down the semiconducting channel. In addition, no band gap is found in the spin-up channel as the alloy in the up-spin channel corresponds to metallic. But on the inclusion of the mBJ scheme, the band gap increases by 3.9 few eV.

**Figure 5:**
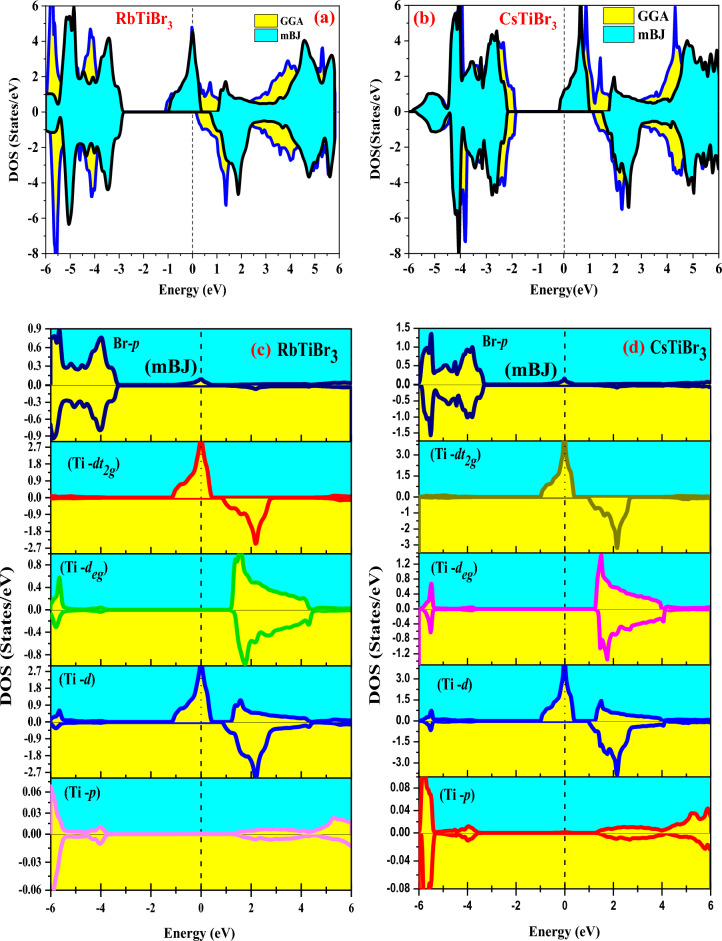
(**a**,**b**) Spin-resolved total density of states (TDOS) of (**a**) RbTiBr_3_ and (**b**) CsTiBr_3_ in GGA and mBJ methods, respectively. (**c**,**d**) Spin-resolved partial density of states (pDOS) of (**c**) RbTiBr_3_ and (**d**) CsTiBr_3_ within mBJ methods, respectively.

Similarly, for the case of CsTiBr_3_, the band gap of 3.4 eV is estimated within the computation of the GGA scheme and 3.8 eV in mBJ. However, the band gap within the approximation scheme of mBJ shows a decreasing trend from Rb to Cs due to an increase in the lattice constant. Further interpretation of their half-metallic nature can be addressed from their corresponding total density of states (TDOS) and partial density of states (pDOS). The total density of states of RbTiBr_3_ and CsTiBr_3_ within GGA and mBJ schemes shows the exact half-metallic nature, as is evident from Fig. 5a,b. Also, the partial density of states within mBJ schemes labeled in Fig. 5c,d illustrates that *Ti-d*t_2g_ pinned at the Fermi level is responsible for showing the metallic nature in spin-up and a semiconducting gap between the *Ti-d*t_2g_ and *Br-p* states in spin-down direction for both the alloys is depicted. So, the overall band structures and their associated density of states results in the half-metallic nature of these two perovskite alloys. The actual half-metallic nature can be understood from the crystal field theory (CFT). From the structural point of view, Ti, the central atom, is caged inside the polyhedrally surrounded by the six neighboring Oxygen atoms, which act as ligands approaching the central metal atom. This kind of approach to ligands lifts the degeneracy of the central atom. Thus, *Ti-d* (*d*_xy_*, d*_yz_*, d*_zx_, *d*_x2_*-d*_y2_, *d*_z2_) gets split into *d*t_2g_ (*d*_xy_*, d*_yz_*, d*_zx_) sets and *d*_eg_ (*d*x^2^*-d*y^2^, *d*z^2^) sets. The *d*t_2g_ states are triply degenerate and have an intake capacity of 6 electrons (3↑, 3↓), while deg states are doubly degenerate and accommodate 4 (2↑, 2↓) electrons.

Meanwhile, the nominal valences of X^+1^Ti^+2^Br^-1^_3_ (X = Rb, Cs) with Ti carry only 2 valence electrons. The possibility of electron filling to form a high spin or low spin state will depend on the crystal field spilling and the type of ligand interacting, either weak or strong. Since the central atom is linked with weak field ligand Br, the splitting will be minor. The filling of electrons will be by the Hund's criterion, i.e., 2t_2g_ (↑), 0e_g_ (↑), 0 t_2g_ (↓), and 0e_g_ (↓) with S = 1 forming the high spin state, thus accordingly featuring the half-metallic nature of these alloys. In addition, the strong-field ligand splitting gap will be higher, and the electron will not prefer to go into the *d*_eg_ orbital. So, the pairing of an electron within the *d*t_2g_ orbital will occur, and hence low spin state will get formed.

### Magnetism and charge density

Next, it is interesting to see the magnetic ground state properties of RbTiBr_3_ and CsTiBr_3_ alloys. For that, different approximation schemes have been taken into consideration. Earlier from the approximation scheme of GGA, the magnetic moment of these two perovskite compounds results in 1.99 μB and 2.00 μB, respectively. It is well known that incorporating GGA often underestimates the electronic structures and magnetic properties. Therefore, staying within the DFT, a modified approximation scheme known as mBJ has been calibrated to identify the accurate magnetic moment corresponding to their lattice structures. By including this potential, it is revealed that the magnetic moment of both the alloys reflects the same as listed in Table 4. The individual magnetic moment of different atoms has been keenly explored. The magnetic moment of Rb and Br is quite feeble, almost equal to zero, and poses its paramagnetic behaviour. Paramagnetic compounds achieve magnetism only when kept in externally applied magnetic fields. However, the negative sign of Br indicates the ferrimagnetic or antiferromagnetic coupling among the other elements taking part within the crystal structure.

**Table 4:** Spin-magnetic moment (µB) of XTiBr_3_ (X = Rb, Cs) compounds.

Magnetic moment (µ_B_)					
Alloys	Rb/Cs	Ti	Br	Interstitial	Total
RbTiBr_3_					
GGA	0.00	1.94	0.01	0.00	1.99
mBJ	0.00	1.97	0.00	0.00	2.00
CsTiBr_3_					
GGA	0.00	1.96	0.01	0.00	2.00
mBJ	0.44	1.97	0.00	0.00	2.00

On the other hand, Ti being the most active and positive, carries its significant magnetic moment towards the lattice structure. Being positive explains the ferromagnetic interaction of the neighbouring atoms. The interstitial magnetic moment has been figured out for both alloys. Therefore, the net magnetic moment arises due to the sum of individual atoms and interstitials. Hence the occurrence of magnetic moment corresponding to their lattice structures lends their possible stand in MRAM applications in spintronics, where magnetic moment rather than electrical charges are used to store information.

**Figure 6:**
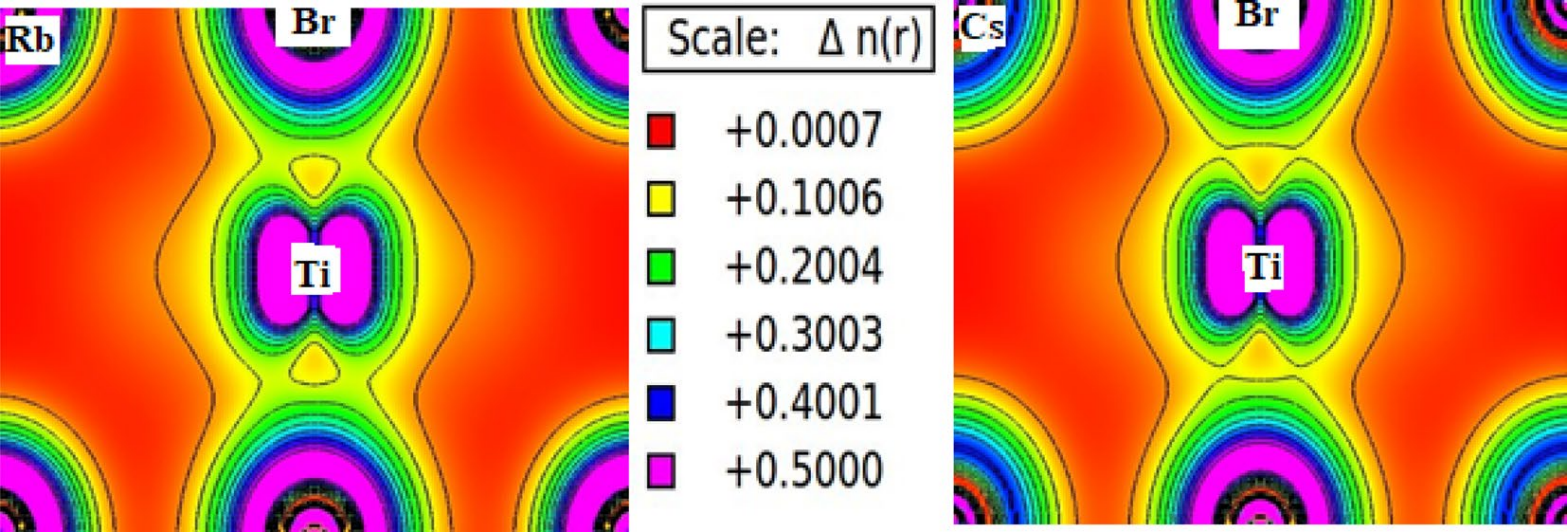
Pictorial representation of electronic charge density of XTiBr_3_ (X = Rb, Cs) in (111) plane.

Next, the interpretation of electronic charge density provides a unique way to understand the chemical stabilities of XTiBr_3_ (X = Rb, Cs) alloys. The spin-polarised charge density plots are presented along the (111) plane as displayed in Fig. 6. We have estimated and shown the bonding characteristics between the various constituents of atoms involved within their lattice structures. The atom that accepts the electrons is electronegative and, simultaneously, which loses are electropositive in character. The same scene is visualised in our case for these systems. The existence of a covalent bond between Ti and Br is properly analysed because the electron cloud of both atoms has non-spherical shapes revealing that the *d* states of Ti are not filled. While the bonding between the Rb/Cs and Br is purely ionic in character by taking its electron cloud own. Therefore, the overall picture reflects the polar covalent bonding, i.e., the admixture of covalent and ionic is preserved within these compounds.

### Thermal properties

**Figure 7:**
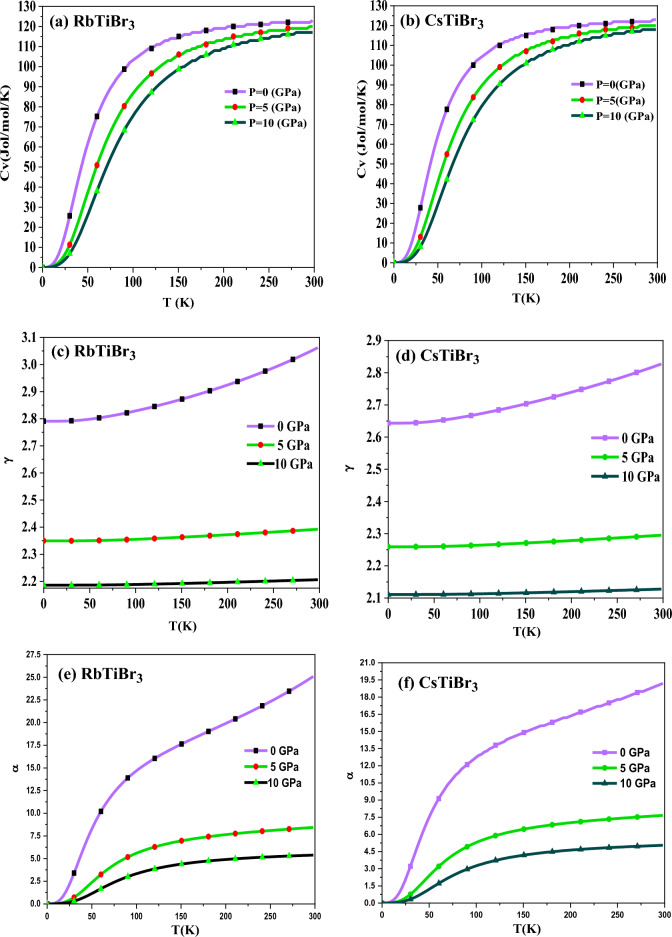

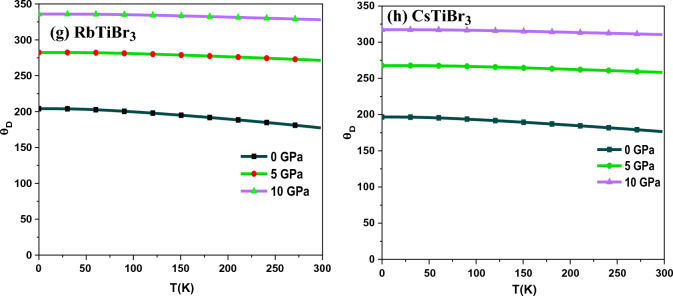
(**a**,**b**) Variation of Specific heat Cv (Jol/mol/K) as a function of temperature. (**c**,**d**) Variation of Gruneisen parameter (γ) as a function of temperature. (**e**,**f**) Variation of thermal expansion (α) as a function of temperature. (**g**,**h**) Variation of Debye temperature (θ_D_) as a function of temperature.

The thermal properties have been carried out within the quasi-harmonic approximation of the Debye model to understand the thermodynamic stability of RbTiBr_3_ and CsTiBr_3_ halide perovskites^31^. Thermal properties assist the essential characteristics of materials, like their nature under temperature and pressure. We have investigated thermal properties like specific heat (C_V_), Gruneisen parameter (γ), thermal expansion (α), and Debye temperature (θ_D_) of these perovskite alloys. These parameters have been keenly evaluated in the pressure and temperature range of “0–10” GPa and “0–300” K, respectively. Nevertheless, remaining under quasi-harmonic approximation, we have illustrated these compounds’ specific heat (C_V_) at constant volume, as shown in Fig. 7a,b. The specific heat of these materials is one of the prime factors that relate to the cause and effect of the dynamics of these materials. The investigated compounds show a remarkable ability of heat with T^3^ relation up to room temperature, experiencing a tendency to follow the Dulong petit limit^42^.

As illustrated in Fig. 7c,d, the Gruneisen parameter (ץ) is explored to see the anharmonicity and provides precise information related to the occurrence of phonon frequency modes. In this investigation, we have noticed that ץ rises as the temperature rises but stays almost constant at higher temperatures. However, the influence of pressure on the Gruneisen parameter has less impact and is almost negligible. At 300 K and 0 GPa pressure, the recorded value of (ץ) for both compounds is 3.05 and 2.83, respectively.

Thermal expansion (α) explains the tendency of these compounds to expand when heat is applied on it. From the Fig. 7e,f, it is seen that α shows a rapid increase up to 60 K, followed by a dull growth for a fixed pressure value for both these alloys. At a specific temperature value, it decreases with increased pressure.

The pressure and temperature dependent Debye temperature (**θ**_**D**_) is depicted in Fig. 7g,h. The Debye temperature is a critical thermodynamic parameter that describes the unique behaviour of these materials. It is clear from these figures that the value of **θ**_**D**_ drops when the temperature rises and increases with the rise in pressure.

### Transport properties

The thermoelectric performance of XTiBr_3_ (X = Rb, Cs) materials can be determined by examining the various distinct transport coefficients such as Seebeck coefficient (S), electrical conductivity (σ/τ), total thermal conductivity (κ), power factor (PF = S^2^σ) and dimensionless figure of merit (ZT) as a function of temperature combined in a compressed form as

8 $${\text{ZT}}\, = \,{\text{S}}^{{2}} \sigma {\text{T}}/\kappa .$$

Where the terms in the mentioned equation have their specific meanings as defined by others elsewhere^46–50^, the transport properties are crucial in understanding the material for its commercial use in thermoelectric applications as it is now clear that the energy crisis is a significant concern due to the vast consumption and contamination of energy resources. Also, the energy is dissipated/released into the environment through residual waste heat. Researchers all over the world are looking to find alternatives for such long-lasting challenges, not just saving energy for today’s generation but for the future also. The scientific community considers this long-term management of energy a hot topic. Because of these situations, we have explored XTiBr_3_ (X = Rb, Cs) alloys because they possess significant half-metallic gaps and a sufficient amount of density of states around the reference level, which promotes us to study the thermoelectric response of these materials. The semiclassical BoltzTraP package^29^ has been used to calculate various thermoelectric coefficients. This program decisively calculates the thermoelectric coefficients such as Seebeck (S), electrical conductivity over the relaxation time (σ/τ), thermal conductivity (κ), power factor (S^2^σ), and figure of merit (ZT).

**Figure 8:**
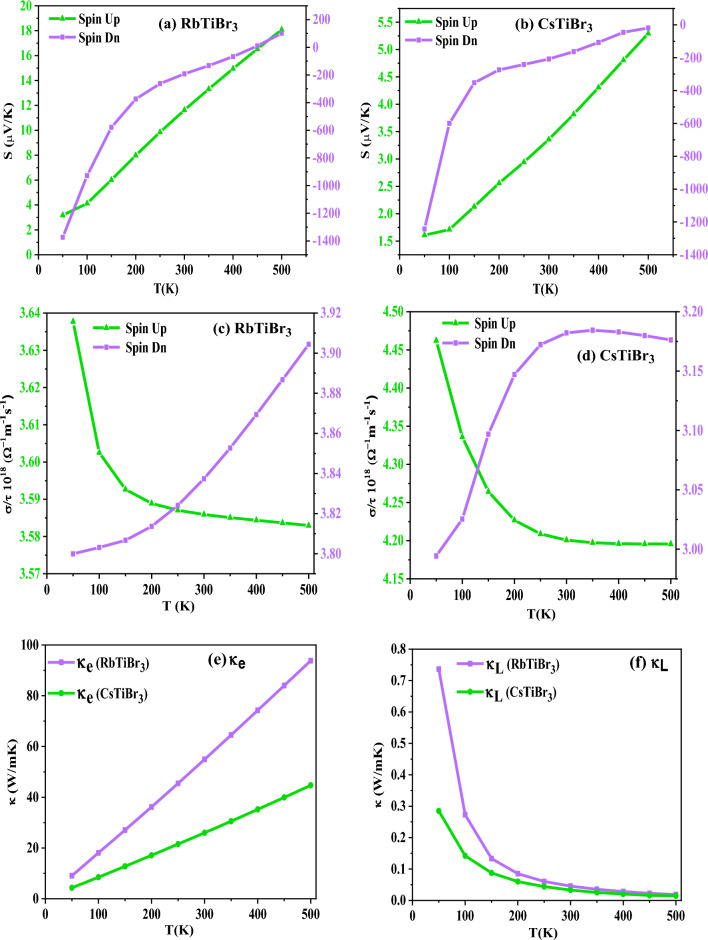

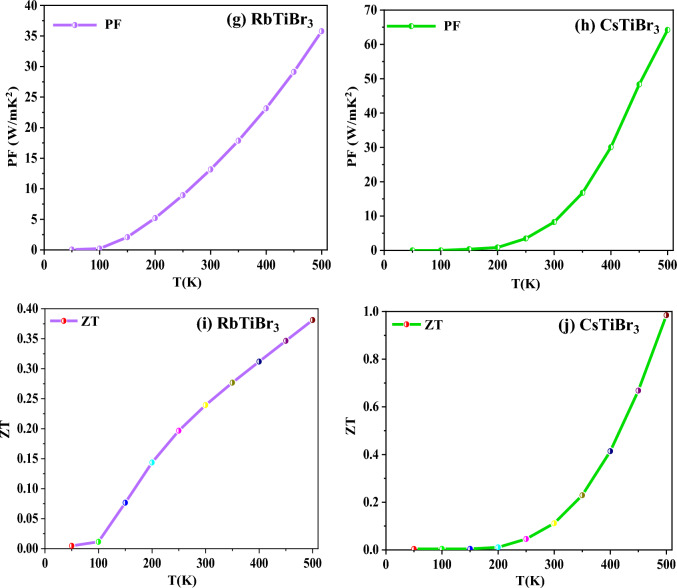
Variation of (**a**,**b**) Seebeck coefficient (S); (**c**,**d**) Electrical conductivity (σ/τ); (**e**,**f**) Total Thermal conductivity (κ); (**g**,**h**) Power factor (S^2^σ); and (**i**,**j**) Figure of merit (ZT) as a function of temperature (K).

Primarily, from the understanding of the Seebeck coefficient (S) describes the ability of a material to generate electrical potential. It also indicates how much voltage gets induced in response to a temperature difference across it. So, keeping this in view, we have examined XTiBr_3_ (X = Rb, Cs) alloys in this report to demonstrate the Seebeck over a specific temperature range. The Seebeck coefficient (S) of both halide alloys has been illustrated and plotted in Fig. 8a,b. The graphical representation of (S) indicates that these alloys follow the absolute trend, which they should follow, i.e., S increases for the spin-up channel as the metallic nature of these materials is convinced and vice-versa, which is true for the half-metallic systems. The perfect increasing nature is seen in the spin-up for the two representative perovskites RbTiBr_3_(CsTiBr_3_) from a low temperature of 50 K with a value of 3.18 µV/K (1.61 µV/K) to a specific value of 18.08 µV/K (5.29 µV/K) at 500 K. In contrast to spin-down, these alloys exhibit semiconducting behaviour. Therefore, S in RbTiBr3 (CsTiBr3) falls significantly from a higher value of − 1373.21 µV/K (− 1243.10 µV/K) to 101.61 µV/K (− 18.59 µV/K) over the selected temperature range. Next, we tried to figure out the electrical conductivity (σ/τ) of a material which defines the concentration of free electrons. In this investigation, we studied the two alloys RbTiBr_3_ (CsTiBr_3_) for their electrical conductivities, as displayed in Fig. 8c,d. From the corresponding spin-up directions, the electrical conductivity (σ/τ) declines exponentially as the temperature rises due to the metallic character and also the positive temperature coefficient of resistance, which possibly figures the decreasing pattern from 3.63 × 10^18^ Ω/m/s (4.46 × 10^18^ Ω/m/s) to 3.58 × 10^18^ Ω/m/s (4.19 × 10^18^ Ω/m/s). However, on the other side, the nature of electrical conductivity exhibits an increasing trend due to its semiconducting nature. The electrical conductivity of RbTiBr_3_ (CsTiBr_3_) increases exponentially from 3.79 × 10^18^ Ω/m/s (2.99 × 10^18^Ω/m/s) to their approximate values of 3.90 × 10^18^Ω/m/s (3.17 × 10^18^Ω/m/s) in the spin-dn channel. The justification can be understood which illustrates that electrons receive enough thermal energy to leap into the conduction band, resulting the formation of electron–hole pairs thus, increases the electrical conductivity. The increasing nature within these halide systems also arises due to the negative temperature coefficient of resistance. Subsequently, thermal conductivity (κ) describes heat transportation through a material as the material’s atoms are constantly moving in rotational, translational, or vibrational motion. The vibration of the atoms is responsible for generating heat or thermal energy in a material. As a result, the process of transporting heat through a material is carried by either electrons or phonons, i.e., the vibration of atoms vibrating at a single frequency known as a phonon. Therefore, the overall contribution of thermal conductivity is the sum of electronic (κ_e_) and lattice (κ_L_), i.e., κ = κ_e_ + κ_L_ as shown in Fig. 8e,f respectively. Individually, Fig. 8e depicts the electronic thermal conductivity (κ_e_) of RbTiBr_3_ (CsTiBr_3_), which displays a growing trend from a low value of 9.07 W/mK (4.33 W/mK) at 50 K to their suitable values of 93.83 W/mK (44.71 W/mK). Furthermore, as descripted in Fig. 8f, we testified these alloys to show their lattice thermal conductivity (κ_L_). We have used the well-known Slacks equation^51,52^ to compute their lattice part. In both these perovskites, the lattice thermal conductivity exhibits a decreasing pattern, ranging from 0.28 W/mK at 50 K to 0.014 W/mK at higher temperatures of 500 K in the case of RbTiBr_3_, whereas CsTiBr_3_ shows a similar trend, ranging from 0.74 W/mK to 0.018 W/mK at the same temperatures. Also, because of this, the enhanced phonon scattering within their lattice structures is quite responsible for decreasing the value of lattice thermal conductivity. Furthermore, it is commonly understood that a material’s power factor (PF) provides electrical energy. A material possessing a high-power factor is a viable candidate for creating more energy. The PF of these respective alloys displays an increasing trend along the designated temperature range, as shown in Fig. 8g,h. The rising trend of PF in RbTiBr_3_ from a negligible value of 0.05 to a higher value of 35.78 W/(mK^2^) can be seen, and similar rising behaviour can be found in CsTiBr_3_ from a small value of 0.25 to a higher value of 64.19 W/(mK^2^). The rising nature of the power factor in both these perovskite systems can be attributed to increased electrical conductivity because the PF is directly related to electrical conductivity (PF = S^2^ σ). Finally, the figure of merit (ZT) values of XTiBr_3_ (X = Rb, Cs) alloys has been examined, as shown in Fig. 8i,j. The value for RbTiBr_3_ exhibits a growing pattern, rising from virtually zero at 50 K to 0.38 at higher temperatures. For CsTiBr_3_, ZT rises and reaches a maximum of 0.98 at 500 K.

### Conclusions

A comprehensive report carried on XTiBr_3_ (X = Rb, Cs) halide perovskites has been summarised by using density functional theory. We conclude that the half-metallic indirect band spectrum of XTiBr_3_ (X = Rb, Cs) is clearly reflected in various approximation schemes. The band-gap of these alloys is found to follow a decreasing trend from Rb to Cs due to an increase in the lattice constant. The magnetic character is the integer value of 2 μB each, thus supporting the half-metallicity within these prescribed alloys. Cohesive energy reveals that CsTiBr_3_ has more attractive surroundings than RbTiBr_3_ requires sufficient energy to liberate an atom from it. In addition, these materials can be used in engineering and other technological applications due to their good mechanical strength. Moreover, phonon dispersions decisively certify the dynamical stability. The calculated Seebeck coefficients, as well as the decent value power factor values of XTiBr_3_ (X = Rb, Cs) and figure of merit are quite satisfactory. The overall theme presents that these perovskite systems' can be used for thermoelectric sources and solid-state device applications like spintronics.

## Data Availability

The data sets analysed or generated during this study will be available from Mr. Vishal Shivhare and Prof. Dinesh C. Gupta upon reasonable request.
